# Trigeminal isolated sensory neuropathy (TISN) and FOSMN syndrome: despite a dissimilar disease course do they share common pathophysiological mechanisms?

**DOI:** 10.1186/s12883-014-0248-2

**Published:** 2014-12-19

**Authors:** Giorgio Cruccu, Elena M Pennisi, Giovanni Antonini, Antonella Biasiotta, Giulia di Stefano, Silvia La Cesa, Caterina Leone, Salvatore Raffa, Claudia Sommer, Andrea Truini

**Affiliations:** Department of Neurology and Psychiatry, Sapienza University, Rome, Italy; Neurology Division, Neurosciences Department, San Filippo Neri Hospital, Rome, Italy; Department NESMOS, Sant’Andrea Hospital, Rome, Italy; Cellular Diagnostics Unit, Department of Clinical and Molecular Medicine, Sant’Andrea Hospital, Rome, Italy; Department of Neurology, University of Würzburg, Würzburg, Germany

**Keywords:** Trigeminal nerve, Neuronopathy, Trigeminal neuropathy, FOSMN, Facial pain

## Abstract

**Background:**

Patients presenting with bilateral trigeminal hypoesthesia may go on to have trigeminal isolated sensory neuropathy, a benign, purely trigeminal neuropathy, or facial-onset sensory motor neuronopathy (FOSMN), a malignant life-threatening condition. No diagnostic criteria can yet differentiate the two conditions at their onset. Nor is it clear whether the two diseases are distinct entities or share common pathophysiological mechanisms.

**Methods:**

Seeking pathophysiological and diagnostic information to distinguish these two conditions at their onset, in this neurophysiological and morphometric study we neurophysiologically assessed function in myelinated and unmyelinated fibres and histologically examined supraorbital nerve biopsy specimens with optic and electron microscopy in 13 consecutive patients with recent onset trigeminal hypoesthesia and pain.

**Results:**

The disease course distinctly differed in the 13 patients. During a mean 10 year follow-up whereas in eight patients the disease remained relatively stable, in the other five it progressed to possibly life-threatening motor disturbances and extra-trigeminal spread. From two to six years elapsed between the first sensory symptoms and the onset of motor disorders. In patients with trigeminal isolated sensory neuropathy (TISN) and in those with FOSMN neurophysiological and histological examination documented a neuronopathy manifesting with trigeminal nerve damage selectively affecting myelinated fibres, but sparing the Ia-fibre-mediated proprioceptive reflex.

**Conclusions:**

Although no clinical diagnostic criteria can distinguish the two conditions at onset, neurophysiological and nerve-biopsy findings specify that in both disorders trigeminal nerve damage manifests as a dissociated neuronopathy affecting myelinated and sparing unmyelinated fibres, thus suggesting similar pathophysiological mechanisms.

## Background

Trigeminal neuropathy is a relatively frequent clinical condition that poses major diagnostic problems to centres treating orofacial pain and headache [1,2]. Although most patients have unilateral trigeminal neuropathy secondary to focal lesions, a few present with a bilateral, symmetric, initially purely sensory trigeminal neuropathy, often related to connective tissue disease or seldom labelled as idiopathic [3,4].

This condition has received attention in sparse case reports, and in three main studies. In the first case series, Spillane and Wells in 1959, described 16 patients with a purely sensory, unilateral or bilateral, trigeminal neuropathy [5]. Several years later, Lecky and colleagues reported 13 patients with idiopathic sensory disturbances restricted to the trigeminal territory, and referred to this condition as “idiopathic trigeminal sensory neuropathy” [3]. More recently, Vucic and colleagues demonstrated that in patients with bilateral trigeminal neuropathy a severe motor involvement might ultimately develop [6,7]. They studied nine patients with bilateral facial-onset sensory deficits in whom, after a mean of 4 years, motor deficits developed and progressed in a rostral-caudal direction. In two autopsy studies, Vucic et al. specified that the disease primarily involved cell bodies in the sensory ganglia and motor nuclei and named this condition “facial onset sensory and motor neuronopathy” (FOSMN) [6-8]. Some reports suggest that FOSMN is a primary neurodegenerative disorder resembling amyotrophic lateral sclerosis, others that it is an immune-mediated neuropathy [8-13].

These studies indicate that a recent onset bilateral trigeminal sensory hypoesthesia may subsequently follow one of two clinical courses: in some patients the disease will remain a trigeminal isolated sensory neuropathy (TISN) in others it will progress to FOSMN, a life-threatening condition manifesting with severe motor involvement.

No diagnostic criteria can differentiate these two conditions. Nor do we know whether they are distinct disease entities or share similar pathophysiological features. Having this information would make it easier to predict a favourable or unfavourable outcome, and help in planning the most appropriate treatment.

Seeking pathophysiological and diagnostic information to distinguish these two conditions, we enrolled 13 consecutive patients presenting with bilateral facial sensory hypoesthesia, did complete neurophysiological trigeminal function testing (assessing all myelinated and unmyelinated fibre groups), and in 10 patients analysed by light and electron microscopy the morphometric features in supraorbital nerve biopsy samples.

## Methods

In the period 1997-2013, in the Policlinico Umberto I and the Ospedale Sant’Andrea (both being University Hospitals of the Sapienza University of Rome), we enrolled 13 consecutive patients with recent onset bilateral trigeminal hypoesthesia and pain (Table 1). Exclusion criteria were connective tissue disease, cognitive disturbances, and other neurological diseases. All patients gave their informed consent to the procedures and the publication of clinical details. The Institutional Review Board of the Policlinico Umberto I – Sapienza University approved the protocol.

**Table 1:** **Clinical data**

**Patient**	**Gender**	**Onset age (years)**	**Onset**	**Duration (years)**	**Clinical course**	**Pain**
**TISN**						
1	F	41	Unilateral paroxysmal pain	20	After 15 years, bilateral ongoing pain and mild sensory deficits.	YES
2	F	55	Bilateral sensory deficit	16	Stable	NO
3	M	57	Unilateral paroxysmal and ongoing pain	13	Slowly developing bilateral sensory deficit and ongoing pain.	YES
4	M	63	Unilateral paroxysmal pain	8	Slowly developing bilateral sensory deficit and ongoing pain.	YES
5	F	23	Unilateral sensory deficit	8	After 5 years bilateral sensory deficit.	NO
6	F	77	Bilateral paroxysmal and ongoing pain	7	Slowly developing bilateral sensory deficit and ongoing pain.	YES
7	M	55	Bilateral sensory deficit	6	After 6 years bilateral sensory deficit.	NO
8	F	63	Unilateral paroxysmal and ongoing pain	6	After 5 years bilateral sensory deficit.	YES
**FOSMN**						
9	M	49	Unilateral ongoing pain	15†	Pain and sensory deficits progressively became bilateral. Motor disturbances began after 6 years. Death 15 years after onset of sensory symptoms.	YES
10	M	50	Bilateral ongoing pain	13	Sensory deficits progressively developed bilaterally. Motor disturbances began after 4 years. In evolution.	YES
11	F	58	Bilateral ongoing pain	8	Sensory deficits progressively developed bilaterally. Motor disturbances began after 2 years. In evolution.	YES
12	F	53	Bilateral sensory deficit	8	Motor disturbances began after 6 years. In evolution.	NO
13	F	56	Unilateral ongoing pain	6†	Pain and sensory deficits progressively became bilateral. Motor disturbances began after 4 years. Death 6 years after onset of sensory symptoms.	YES

*TISN:* Trigeminal isolated sensory neuropathy. *FOSMN:* Facial onset sensory-motor neuronopathy. †deceased.

### Clinical, neuroimaging and laboratory investigations

All patients underwent a detailed neurological examination using bedside tools. Trigeminal and extra-trigeminal sensory function were assessed: touch was investigated with a piece of cotton wool, vibration with a tuning fork (128 Hz), and pinprick sensation with a wooden cocktail stick. Gait impairment, and muscle strength were assessed with the Medical Research Council score. Patients were also asked to report dysautonomic symptoms.

All patients underwent laboratory testing, including tests to exclude identifiable causes of trigeminal neuropathy: autoantibody essays to detect connective tissue disease (antinuclear antibodies, anti-double-stranded DNA, antinuclear extractable antigens, including anti Sm, anti RNP, anti Scl70, and anti-phospholipids, antineutrophil cytoplasmic antibodies and anti Ro/SSA and anti-La/SSB for Sjögren’s disease). Some patients also underwent the genetic serum test for Kennedy’s disease, cholesteryl esters and low serum cholesterol for Tangier disease, glycosphingolipid accumulation for Fabry’s disease, and serum-angiotensin converting enzyme for neurosarcoidosis. All patients underwent brain and spinal cord gadolinium-enhanced magnetic resonance imaging (MRI).

### Trigeminal neurophysiology

We tested trigeminal motor evoked potentials after transcranial magnetic stimulation [14], the temporalis H reflex, assessing Aα fibre (Ia fibre) in the monosynaptic trigeminal reflex [15]. We also tested the early blink reflex components (R1) after electrical supraorbital nerve stimulation and the masseter inhibitory reflex (SP1) after mental nerve stimulation, assessing Aβ fibres [16]. We recorded laser evoked potentials (LEPs) to investigate Aδ nociceptors (Aδ-LEPs) and unmyelinated warmth receptors (C-LEPs) [17].

All patients underwent nerve conduction studies (NCS) using surface recording electrodes placed in the standard manner. We recorded sensory nerve action potentials (SNAPs) and conduction velocities from sural, ulnar and superficial radial nerves. Other nerve function variables examined were compound motor action potential (CMAP) amplitude and peroneal, tibial and ulnar nerve conduction velocities. Electromyographic (EMG) investigation included pontobulbar muscles (orbicularis oris muscle, genioglossus muscle, sternocleidomastoid muscle, masseter muscle), limb muscles (biceps brachii, extensor digitorum communis, first dorsal interosseus, lateral vastus, tibialis anterior), and cervical paraspinal muscles.

Neurophysiological testing adhered to the technical requirements issued by the International Federation of Clinical Neurophysiology [18,19].

### Nerve biopsy and nerve morphometry

Supraorbital nerve biopsies were performed by a trained plastic surgeon, in a period ranging between 3 and 6 years from beginning of disease. Specimens fixed with 2% glutaraldehyde in phosphate-buffered saline (PBS) at 4°C. Samples were post-fixed in 1% osmium tetroxide in veronal acetate buffer (pH 7.4) for 1 h at 25°C, stained with uranyl acetate (5 mg/ml) for 1 h at 25°C, dehydrated in acetone and embedded in Epon 812 (EMbed 812, Electron Microscopy Science, Hatfield, PA, USA). For each sample, semithin sections were stained with toluidine blue for light microscopy assessment. Ultrathin sections from tissue blocks with the proper orientation, post-stained with uranyl acetate and lead hydroxide, were examined under a Morgagni 268D transmission electron microscope (FEI, Hillsboro, OR, USA). For nerve morphometry a total of 20 different microscopic fields, randomly taken from ultrathin sections from all the available fascicles, were acquired at 28,000 X original magnification and digitalized with a Mega View II charge-coupled device camera (SIS, Soft Imaging System GmbH, Munster, Germany). The digital images were analyzed with AnalySIS software (SIS) and all myelinated and unmyelinated structures were identified and measured. Fibre densities were calculated and expressed as the mean number of fibres/mm^2^; fibre size distributions were represented in histograms.

**Figure 1:**
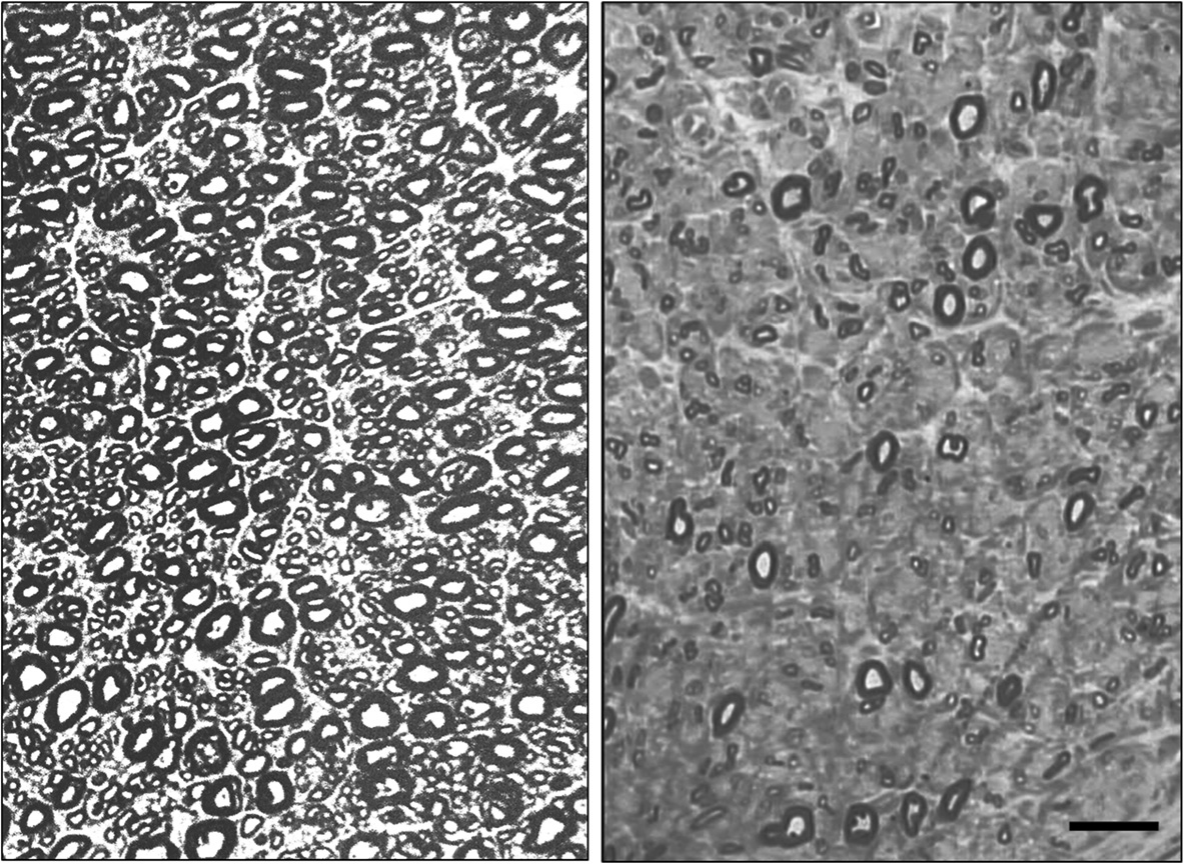
**Light microscopy.** Photomicrographs of semithin sections. left figure: normal supraorbital nerve from patient 0. Note the densely packed myelinated fibres. right figure: supraorbital nerve from Patient 5 showing rarefied large-size myelinated fibres, whereas some small myelinated fibres are preserved. Bar: 20 μm.

Morphometric data in patients with TISN and FOSMN, were compared with supraorbital nerve findings in a 73-year-old woman (Patient 0, Table 1, Figure 1) who had a 10-year history of constant burning pain in the face. The pain started in the nose and left cheek, and then progressively spread to involve the mouth and lips bilaterally. On sensory examination, the patient reported hypoesthesia on the skin of the nose and upper lips. A computed tomographic (CT) scan and two MRI scans showed no abnormalities consistent with her pain. To exclude trigeminal neuropathy causing supraorbital nerve damage even in a patient reporting sensory disturbances only in the lower face we proposed a supraorbital nerve biopsy. Examination of the nerve biopsy specimen disclosed no abnormality, myelinated fibres were bimodally distributed peaking at 4.5 and 11 μm and their density was about 10,000/mm^2^, whereas unmyelinated fibres had a unimodal distribution peaking at 0.8 μm and their density was about 40,000/mm^2^. A third MRI scan eventually disclosed abnormal tissue in the left maxillary sinus. After surgery, histological examination demonstrated a well-differentiated Malpighian cancer. We concluded that the tumor had slowly invaded the infraorbital canal, thus explaining the sensory disturbances and neuropathic pain on the left side. After lengthy discussion, we tentatively attributed the contralateral disturbances either to central sensitization involving the second-order neurons that receive bilateral input from the deep trigeminal territories or to a psychological component.

All the morphometric results had a normal distribution. Differences in normally-distributed morphometric measures were evaluated with t test for unpaired data.

## Results

### Clinical findings

In eight patients (follow-up 10.5 ± 5.2 years), clinical disturbances remained restricted to trigeminal sensory hypoesthesia and pain without progressing to FOSMN (Table 1). The patients’ mean age at onset was 54 ± 16 years. In some patients symptoms began with unilateral paroxysmal pain, described as electric shock-like sensations that resembled classic trigeminal neuralgia (Table 1). In these patients paroxysmal pain progressively disappeared as the sensory deficits slowly developed and eventually stabilized. Three patients (patients 2, 5, and 7) had sensory deficits alone. In all 8 patients repeated NCS and needle electromyography excluded subclinical extra-trigeminal spread.

Conversely, in five patients (follow up 10.0 ± 3.8 years) the condition spread to cranial and limb motor nerves, manifesting with FOSMN. Their mean age at onset was 53 ± 4 years, similar to that in the group with the isolate trigeminal neuropathy, whereas the age range was narrower, varying from 49 to 58 years (Table 1). The onset symptoms and disease course were also similar, except that no patient in this group had paroxysmal pain. Within a few years (range 2-6 years) four patients manifested dysarthria and dysphagia, whereas one reported masticatory weakness followed by asymmetric facial paresis. In all five patients, the motor deficit rapidly involved other cranial nerves but spared the oculomotor nerves. Patient 9 died 15 years after onset. Patient 13, notwithstanding attempted therapy with intravenous immunoglobulins, died 6 years after onset. In the other three patients, we tried intravenous immunoglobulins: two patients reported a subjective benefit, but neither their clinical nor their neurophysiological status improved.

### Neurophysiological investigations

In both groups trigeminal motor evoked potential recordings and temporalis H-reflex testing yielded normal findings; conversely trigeminal reflex recordings showed severe trigeminal abnormalities: the first response to become absent bilaterally was the early (SP1) masseter inhibitory reflex after mental nerve stimulation. The early (R1) blink reflex, however, was often delayed or even absent before the patient noticed a sensory disturbance in the ophthalmic division. Whereas the Aδ-fibre mediated LEPs were frequently abnormal (but less impaired than the early trigeminal reflexes), the C-LEPs, mediated by unmyelinated C-fibres^11^ were normal in all patients in both groups.

These neurophysiological abnormality patterns in general suggested disease progressing from the largest to the smallest afferent fibres. The one noteworthy exception was the normal Aα afferent-mediated temporalis H reflex in all patients in both groups.

Whereas in patients with trigeminal isolated sensory neuropathy NCS and EMG disclosed unremarkable findings, in patients with FOSMN NCS showed slightly reduced ulnar and radial SNAPs in four patients, and EMG showed denervation–reinnervation changes (large amplitude, long-duration polyphasic motor unit potentials), fibrillation potentials, sharp waves and fasciculation in all patients.

### Histological findings from nerve biopsy

In all patients the supraorbital nerve biopsy did not produce any esthetical damage or other complaint.

**Figure 2:**
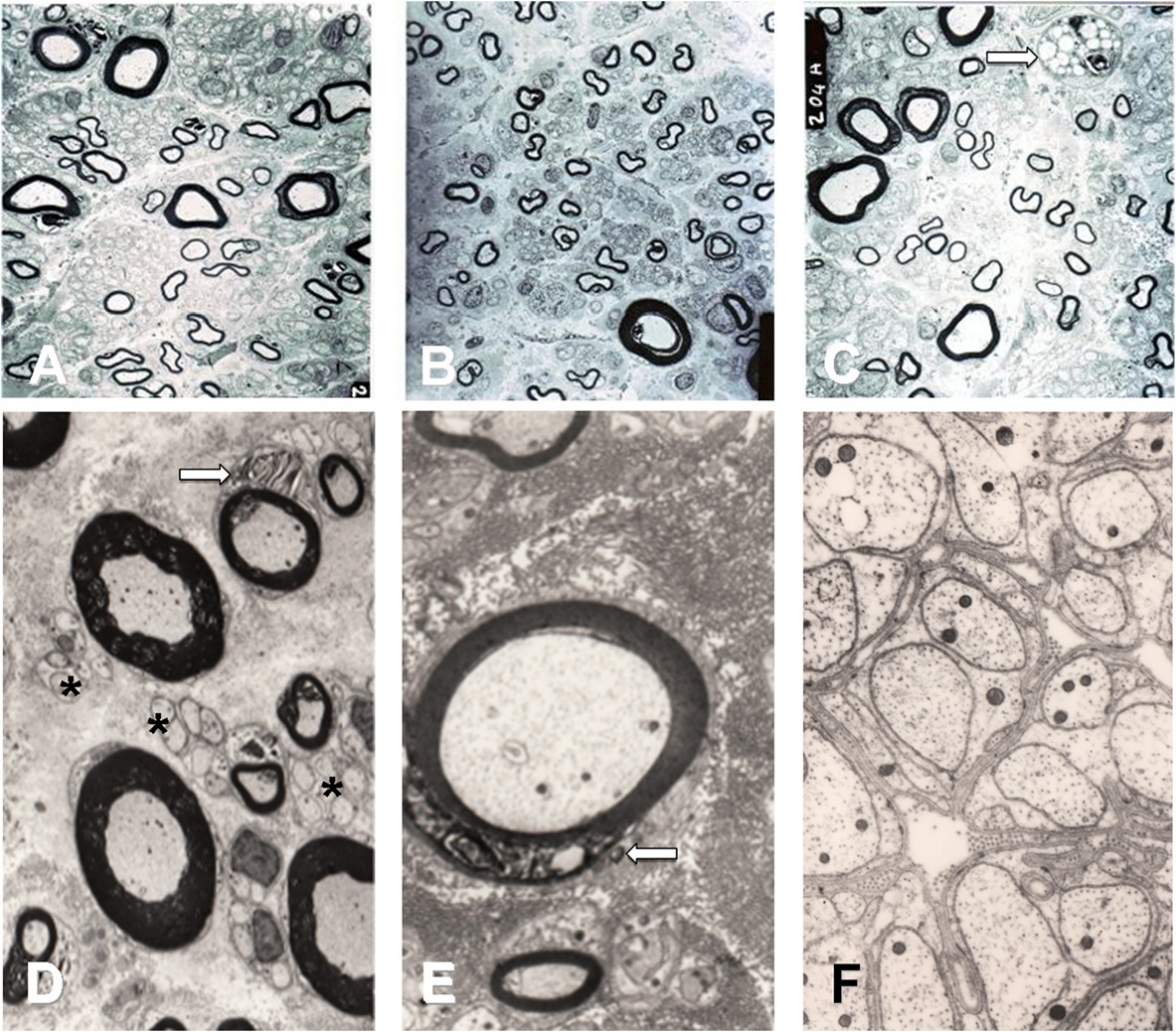
**Electron microscopy.** Upper panel: low-power electron micrographs taken on ultrathin sections from the supraorbital nerve in Patients 2 **(A)**, 3 **(B)** and 11 **(C)**. Bar: 10 μm. The supraorbital nerve contains remarkably fewer large myelinated than small myelinated fibres. The macrophage in C (arrow) indicates axonal breakdown and phagocytosis. Lower panel: high-power electron micrographs taken on ultrathin sections from the supraorbital nerve in Patient 6 **(D,E)** and Patient 9 **(F)**. Bar: 10 μm in D, 5 μm in E, and 1 μm in F. Myelin debris in Schwann cells (arrows), indicating myelin degradation in D and E. The asterisks in F and D indicate intact unmyelinated fibres.

**Figure 3:**
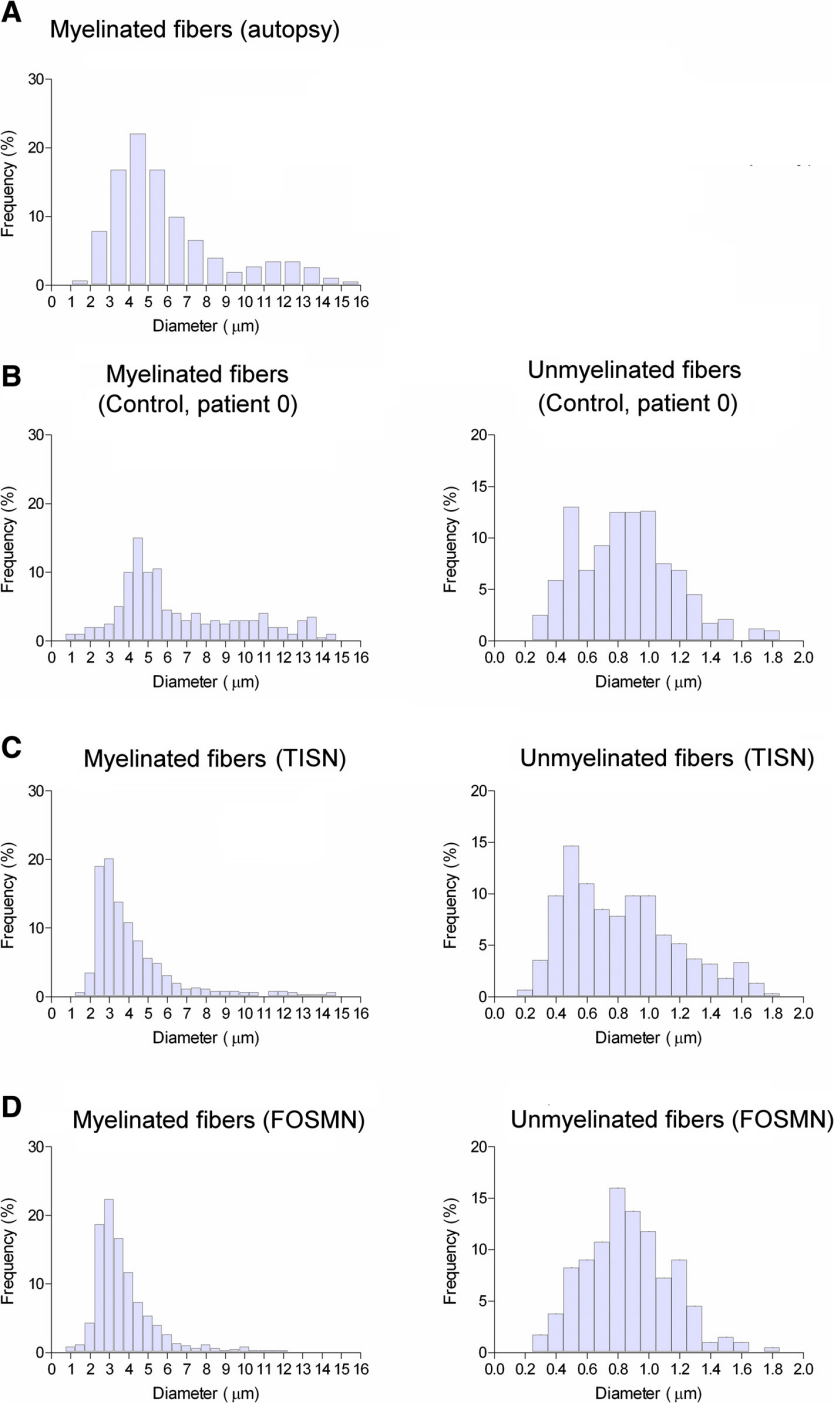
**Histograms.** Histograms for myelinated (left) and unmyelinated (right) fibres. **A**: grand average for three autopsy specimens (Pennisi et al. [20]). **B**: Patient 0. **C**: grand average for six patients with trigeminal isolated sensory neuropathy (TISN). **D**: grand average for four patients with facial-onset sensory motor neuronopathy (FOSMN). Myelinated fibres in patients with trigeminal neuronopathy are unimodally distributed and the two conditions yield similar histograms.

In biopsy specimens from the two groups of patients, histological examination yielded almost matching findings: both light and electron microscopy showed only a Wallerian-like degeneration involving myelinated fibres, more severe for the large Aβ- than for the small Aδ-fibre group, with no inflammatory changes (Figures 2 and 3). The histograms for myelinated fibres in both groups therefore showed a unimodal distribution with loss of the larger peak (Figure 3). Similarly, the qualitative and quantitative measures yielded almost matching fibre density, peak, and maximum diameter (Table 2).

**Table 2:** **Morphometric data**

	**Myelinated fibres**			**Unmyelinated fibres**		
**Patient**	**Density (n/mm** ^**2**^ **)**	**Peak (μm)**	**Max (μm)**	**Density (n/mm** ^**2**^ **)**	**Peak (μm)**	**Max (μm)**
0	10000	4.5-11	14.5	40000	0.8	1.8
**TISN**						
1	-					
2	4500	5	13	30000	1.0	1.7
3	5000	5	14	35000	0.9	1.5
4	-					
5	3212	2	14	43210	1.0	1.8
6	2526	3	8	52751	1.1	1.6
7	12073	3	11	36997	0.8	1.4
8	5526	3.5	11	50600	0.8	1.7
Mean ± SD	5473 ± 3421	3.6 ± 1.2	11.8 ± 2.3	41426 ± 9023	0.9 ± 0.1	1.6 ± 0.1
**FOSMN**						
9	2500	4	8	40000	1.0	1.7
10	1150	3.5	12.5	31600	0.9	1.6
11	7829	2.5	10	54197	0.8	1.4
12	-					
13	11639	3	11.5	59978	1.0	1.8
Mean ± SD	5780 ± 4855	3.3 ± 0.6	10.5 ± 2.0	46444 ± 12976	0.9 ± 0.1	1.6 ± 0.2
P	>0.90	>0.60	>0.35	>0.45	>0.90	>0.90

*TISN*: Trigeminal isolated sensory neuropathy. *FOSMN*: Facial onset sensory-motor neuronopathy. P: t test between TISN and FOSMN.

Electron microscopy of unmyelinated fibres disclosed no collagen pockets or other abnormalities. The histograms resembled those from sural nerve biopsy. Morphometric data were also similar in both groups (Table 2 and Figure 3).

## Discussion

Despite detailed neurophysiological and morphometric investigations we found no clinical, neurophysiological or neuropathological differences that could differentiate between TISN and FOSMN. Hence, we conjecture that the two diseases might be pathophysiologically similar neuropathies. Our study extends current knowledge [3-7] by showing that TISN and FOSMN are dissociated neuronopathies that fully spare unmyelinated fibres.

### A dissociated neuropathy

When we quantitatively assessed trigeminal nerve fibres, a distinctive feature in this study, the neurophysiological data from 13 patients and morphometric findings from 10 supraorbital nerve biopsies demonstrated that both diseases, TISN and FOSMN, fully spared trigeminal unmyelinated fibres (Table 1).

Light and electron microscopy in supraorbital nerve biopsy specimens from patients with TISN and those with FOSMN showed a variably severe axonal myelinated fibre loss, as others have reported in these patients [3,6]. We extend these findings by providing quantitative data showing that trigeminal neuropathy affects Aβ- more severely than Aδ-fibres. The most striking feature in the histograms for myelinated fibres was the unimodal distribution reflecting the marked reduction in or loss of the larger peak. Normal trigeminal sensory nerves have a bimodal distribution, similar to that for the sural nerve, as seen in Patient 0 and demonstrated in autopsy specimens from three healthy subjects [20]. Evidence that nerve fibre damage progresses from the largest to the smallest fibre comes also from the neurophysiological findings, invariably showing impaired Aβ-fibre-mediated responses even at the earliest disease stages. Conversely, Aδ-fibre-mediated responses were far less impaired.

### A neuronopathy

In patients with TISN and in those with FOSMN the temporalis H-reflex sparing provides evidence that these two conditions primarily affect cell bodies [6,7]. A dissociated neuropathy that progressively affects the largest then the smallest myelinated fibres should in theory severely alter a reflex mediated by Aα afferents from muscle spindles. Conversely, it spares the primary afferents from trigeminal muscle spindles because they travel in the motor rather than the sensory root. Equally important, rather than lying in the sensory ganglion their cell bodies lie in the mesencephalic trigeminal nucleus [21,22]. This unique anatomic feature also explains why the mandibular tendon jerk (or jaw jerk) is spared in two other trigeminal neuronopathies: Sjögren’s syndrome and Kennedy’s disease [23,24].

Unlike previous studies that used a hand-held reflex hammer to elicit the mandibular tendon jerk we used the temporalis H reflex to avoid possible interference from temporomandibular dysfunction or malocclusion, conditions that can induce abnormalities or even an absent reflex response to the hand-held reflex hammer [25].

### Differential diagnosis

The variable clinical presentation at the onset of bilateral trigeminal neuropathy makes the differential diagnosis with the various possible trigeminal nerve diseases a challenging task [26]. Several patients in our series had unilateral paroxysmal pain as the first symptom. Hence, a few patients might be initially diagnosed as having trigeminal neuralgia. This diagnostic error can be avoided by routinely including trigeminal reflex testing in the diagnostic work-up. According to European and American guidelines, the early (Aβ-mediated) responses are normal in over 90% of patients with classic trigeminal neuralgia, whereas in patients with trigeminal neuropathy they are always abnormal [27,28].

Bilateral trigeminal neuropathy is often related to connective tissue disease [4]. Trigeminal neuropathy sometimes heralds the onset of systemic sclerosis, mixed connective tissue disease, or Sjögren's syndrome [29]. Whereas Sjøgren’s syndrome is easily recognised by its other typical symptoms, xerophthalmia and xerostomia, other connective tissue diseases may be more difficult to differentiate at an early stage.

A condition that closely resembles the FOSMN syndrome is the Kennedy’s disease, an X-linked hereditary disease that progressively affects primary sensory neurons and motoneurons [30]. The onset can occasionally be trigeminal, and thus male patients with this type of trigeminal neuronopathy must undergo genetic testing for Kennedy’s disease.

All the other diseases in the differential diagnosis, such bulbar-onset amyotrophic lateral sclerosis, the various forms of Tangier and Fabry’s disease, neurosarcoidosis, and syringobulbia, are easily ruled out by clinical history and examination [3,6,7,31].

### Pain mechanisms

In our patients with TISN and FOSMN, the dissociated nerve fibre involvement should provide relatively sound information about how specific nerve-fibre groups intervene in the pathophysiology of the various types of neuropathic pains [32], in particular ongoing pain due to deafferentation. Our findings on pain nevertheless go against classic notions about pain mechanisms. First, histological findings and neurophysiological testing showed that the unmyelinated fibres were fully spared. Secondly, during the disease course, as the sensory deficit progressively increased in severity, i.e. more and more primary neurons degenerated, the pain tended to disappear. These findings clearly argue against spontaneous hyperactivity in deafferented nociceptive second-order neurons [32]. A possible explanation for these contrasting results on the relationship between myelinated nerve fibres and the development of neuropathic pain lies in the imbalanced input from myelinated and unmyelinated nerve fibres onto the second-order neurons [33].

## Conclusions

Because TISN and FOSMN share almost identical clinical, neurophysiological and morphometric trigeminal features at onset, no currently available diagnostic techniques can predict their clinical course. Nor can we predict whether patients with bilateral trigeminal sensory hypoesthesia and pain will ultimately have FOSMN. In TISN as well as in FOSMN, trigeminal nerve damage manifests as a dissociated neuronopathy affecting myelinated and sparing unmyelinated fibres. Although we cannot conclude that TISN and FOSMN are the same disease, given that the two conditions cause similar trigeminal nerve damage they might also share similar pathogenetic mechanisms. In this case, the different course of disease might be explained—as a few patients were reported to respond to immunomodulating drugs [11,12]—by dysimmune mecanisms with different targets in TISN and FOSMN.

Those who work in centres specialized in orofacial pain or headache should be aware that a patient who initially manifests sensory disturbances on one side alone may later go on to manifest trigeminal bilateral neuronopathy. Hence, they should refer patients who begin to experience contralateral sensory symptoms for detailed diagnostic investigations. Although no therapy is currently effective, an early diagnosis would inform the patient about the outcome and exclude other possibly treatable causes.

## Abbreviations

CMAP: Compound Motor Action Potential
EMG: Electromyography
FOSMN: Facial Onset Sensory Motor Neuronopathy
NCS: Nerve Conduction Study
LEP: Laser Evoked Potentials
SNAP: Sensory Nerve Action Potential
TISN: Trigeminal Isolated Sensory Neuropathy
